# Evaluation of Compressive Strength and Stiffness of Grouted Soils by Using Elastic Waves

**DOI:** 10.1155/2014/215804

**Published:** 2014-06-12

**Authors:** In-Mo Lee, Jong-Sun Kim, Hyung-Koo Yoon, Jong-Sub Lee

**Affiliations:** ^1^School of Civil, Environmental and Architectural Engineering, Korea University, Seoul 136-701, Republic of Korea; ^2^Department of Geotechnical Disaster Prevention Engineering, Daejeon University, Daejeon 300-716, Republic of Korea

## Abstract

Cement grouted soils, which consist of particulate soil media and cementation agents, have been widely used for the improvement of the strength and stiffness of weak ground and for the prevention of the leakage of ground water. The strength, elastic modulus, and Poisson's ratio of grouted soils have been determined by classical destructive methods. However, the performance of grouted soils depends on several parameters such as the distribution of particle size of the particulate soil media, grouting pressure, curing time, curing method, and ground water flow. In this study, elastic wave velocities are used to estimate the strength and elastic modulus, which are generally obtained by classical strength tests. Nondestructive tests by using elastic waves at small strain are conducted before and during classical strength tests at large strain. The test results are compared to identify correlations between the elastic wave velocity measured at small strain and strength and stiffness measured at large strain. The test results show that the strength and stiffness have exponential relationship with elastic wave velocities. This study demonstrates that nondestructive methods by using elastic waves may significantly improve the strength and stiffness evaluation processes of grouted soils.

## 1. Introduction

Cement-based soil-grout mixtures (grouted soils) have been commonly used during the construction of underground spaces for the improvement of the strength and stiffness of soils and rocks and for the prevention of the leakage of ground water. A practical cement-based grouting technique, however, is not available for penetrating into the small-sized pore geomaterials. Furthermore, the curing of grouting takes 2~3 days. Thus, fine to micro-fine cement grains have been developed in order to improve groutability [1]. Quick setting agents have been used to control gel-time or to cure the grouting within a few minutes.

The curing time and initial strength of grouted soils play a crucial role in ground improvement. Ground improvements, however, have been commonly evaluated by the destructive method in both the field (in situ) and the laboratory. In situ methods include the standard penetration test (SPT) and the cone penetration test (CPT). Laboratory methods include the uniaxial compression test, the triaxial compression test, and the direct shear test. The sensitivity of SPT and CPT, however, is not sufficient for the evaluation of ground improvements. Furthermore, the sampling for the laboratory test yields a disturbance. In the field, the weak parts are not generally obtained during the coring or drilling of soil-grout mixtures. The strength, which is based on the laboratory tests, may therefore be slightly overestimated. To overcome the numerous limitations of the destructive strength test, a nondestructive test using elastic waves may be an alternative option. Nondestructive tests based on elastic waves include the transmission and reflection methods. Note that the penetration types of elastic wave measurement techniques were also suggested [2–4]. In this study, the transmission method was used, which uses two transducers on the two opposing faces of the specimen.

The goal of this study is to estimate the uniaxial compressive strength and elastic modulus of the grouted soils with and without grains (particulate materials) by using elastic waves. This paper includes specimen preparation, the measurement system, stress-strain responses during the uniaxial compression test, compressional and shear wave responses under applied uniaxial stress, and correlations between strength or stiffness and elastic wave velocities.

## 2. Elastic Wave Velocity versus Strength and Stiffness

The strength and stiffness of concrete may be empirically determined from nondestructive methods by using elastic waves. Although the theoretical relationship between the elastic wave velocity and the strength or elastic modulus cannot be derived in a composite material, attempts have been made over the past sixty or more years to correlate the properties of composite materials with elastic wave velocities [5]. The strength of high strength concrete has been obtained based on nondestructive methods [6]. Similarly, the strength and elastic modulus of concrete have been obtained by using elastic wave velocities. The relationships between the strength or elastic modulus and elastic wave velocity are based on empirical correlations (exponential functions) as follows:

(1) $$\begin{array}{c} f_{c}=\alpha e^{\beta V_{P}}, \end{array}$$

(2) $$\begin{array}{c} E=ae^{bV_{P}}, \end{array}$$

where *f*_*c*_ is the uniaxial compressive strength, *E* is the elastic modulus, *V*_*P*_ is the compressional wave velocity, and *α*, *β*, *a*, and *b* are coefficients determined by experimental results. Equations (1) and (2) have been used in concrete [5, 7, 8]. The empirical Equation (1) may be rewritten in the form of the bilinear function by using a log scale in the *f*_*c*_-axis [5]. For soil-grout mixtures, since an empirical correlation has not been suggested, a relationship is required for the estimation of the strength of soil-grout mixtures.

## 3. Experimental Study

Nondestructive tests by using elastic waves were conducted before and during the axial compression test. The material properties, preparation of specimens, and measurement systems are described as follows.

### 3.1. Material Properties

**Figure 1:**
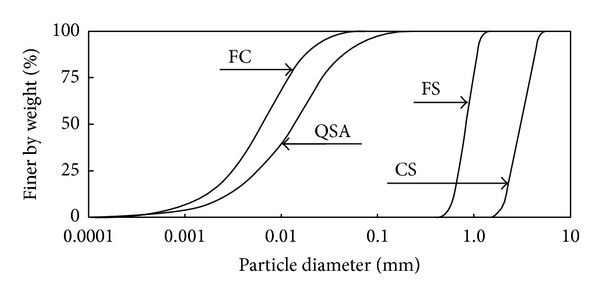
Grain-size distribution of coarse and fine grains, cement, and quick setting agent. FC, QSA, FS, and CS denote fine cement, quick setting agent, fine sands, and coarse sands, respectively.

**Figure 2:**
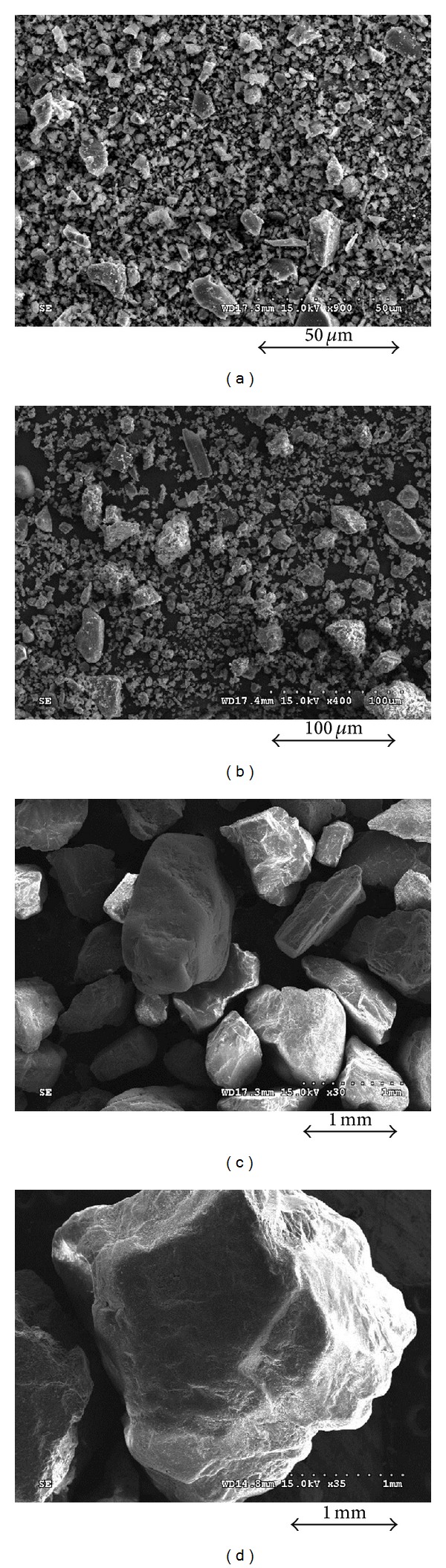
Photomicrographs: (a) fine cement; (b) quick setting agent; (c) fine sands; (d) coarse sands.

The cemented grouted soils comprised of two types of uniform sands, fine particle cements, and quick setting agents. The grain size distribution curves and photographic images of the materials are plotted in Figures 1 and 2. Two types of sands were used: fine and coarse sands. The physical properties of the two sands are summarized in Table 1. The median diameters were 0.82 mm and 3.1 mm for fine and coarse sands, respectively. Table 1 shows that the friction angle obtained from the direct shear tests was smaller in fine sands than in coarse sands. The coefficient of uniformity, coefficient of curvature, and specific gravity in both sands were similar to each other.

**Figure 3:**
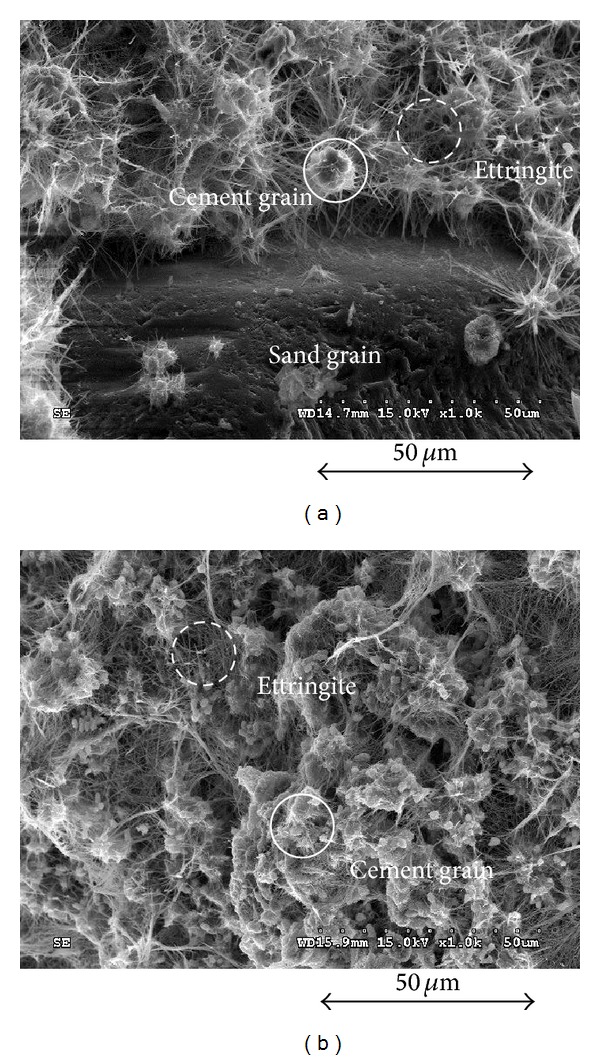
SEM images of soil-grout mixtures: (a) specimen with sands; (b) specimen without sands.

The median diameters of fine cement and quick setting agents were 6.6 *μ*m and 17 *μ*m, respectively, as shown in Figure 1. The component of fine particle cements was similar to Portland cements except for the average particle size (35 *μ*m). The quick setting agent is an additive for the hydration of cement. The chemical reaction between the fine particle cement and quick setting agent dramatically increases the particle concentration and viscosity of the mixture after mixing due to the generation of ettringite (hydrated calcium aluminum sulfate hydroxide), thaumasite, and alite (tricalcium silicate). Note that ettringite, which was produced during the early hydration of cement [9], was activated by the quick setting agent. The SEM images of mixtures with and without sand grains are shown in Figure 3. Figure 3 shows that sand and cement particles were connected by ettringite. In addition, the mixture with fine cement and quick setting agent produced a relatively larger void ratio and lower density than a regular cement-based soil-grout mixture without a quick setting agent. In addition, because the mixture was hardened before the complete drainage of free water, the strength and elastic wave velocity of the mixture would be lower.

### 3.2. Preparation of Grout Soils

Five types of the grouted soils without sand grains (P-type), three types of the soil-grout mixtures with fine grain sands (F-type), and three types of the soil-grout mixtures with coarse grain sands (C-type) were prepared to control the uniaxial compressive strength at large strain and elastic wave velocities at small strain. Details of soil-grout mixtures are presented in Table 2 including mixing ratio, water-cement ratio, unit weight of the specimens, and number of specimens. Two to four specimens were prepared in plastic molds for each type, with a total of thirty-four specimens. The diameter and length of each specimen were 45 mm and 90 mm, respectively. The specimens were cured underwater for 7 days.

### 3.3. Compressive Strength Measurements

**Figure 4:**
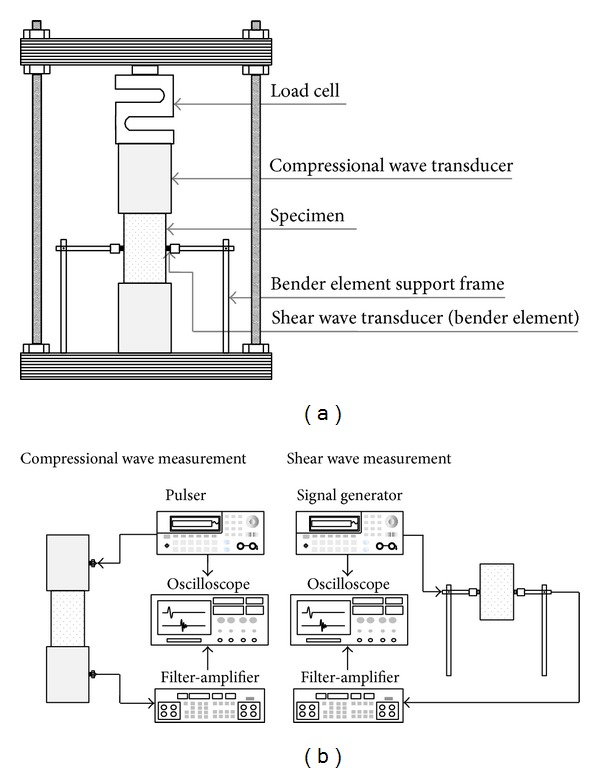
Experimental setup: (a) unconfined axial compression test; (b) elastic wave measurements.

The compressive strength was measured by an unconfined axial compression test as shown in Figure 4. The vertical strain rate was 0.001/sec. Three linear variable differential transformers (LVDT) were adopted to measure axial and horizontal deformations. One load cell was used to obtain the axial load. The responses of three LVDTs and one load cell were monitored by the data logger (Agilent 34970A) as shown in Figure 4.

### 3.4. Elastic Waves Measurement

The compressional waves were measured by compressional wave transducers during an axial compression test as shown in Figure 4. The diameter of the transducers was 50 mm, which corresponds to the diameter of the specimens. The very strong compression wave transducers were installed on the top and bottom of the specimen. Therefore, the axial load was directly applied through the transducers into the specimen. The resonant frequency of the transducers was 50 kHz. The impulse signal, which was generated by the pulser (JSR DPR 300), activated the source transducer. The excitation of the source transducer yielded compressional waves (P-waves). The P-waves were propagated through the specimen, detected by the receiver transducer, and were recorded by the digital oscilloscope (Agilent 54624A). Note that the compressional waves were measured by the pulse velocity method, which was specified in ASTM C597 and C1383 [10, 11]. The details in P-wave measurement were discussed in Lee and Santamarina [12] and Lee et al. [13].

The measurement system of the shear waves was similar to that of the compressional waves. The shear waves were measured by bender elements during an axial compression test as shown in Figure 4. The cantilever length and the resonant frequency of the bender elements were 2 mm and 5 kHz, respectively. The experimental setup for the measurement of shear waves was described in Lee and Santamarina [14, 15] and Lee et al. [16]. The bender elements were installed on the sides of the specimen by using a bender supporter frame as shown in Figure 4. For the excitation of the bender elements, square waves with 10 V in peak-to-peak amplitude were continuously generated by the signal generator (Agilent 33220A). The shear wave responses were measured by the digital oscilloscope (Agilent 54624A) as shown in Figure 4. The details in the bender elements were described in Lee and Santamarina [14].

## 4. Experimental Results

After all specimens were cured for 7 days after casting, the compressive strength tests were carried out. The responses of elastic waves during axial compression tests were also monitored.

### 4.1. Stress-Strain Responses at Large Strain

**Figure 5:**
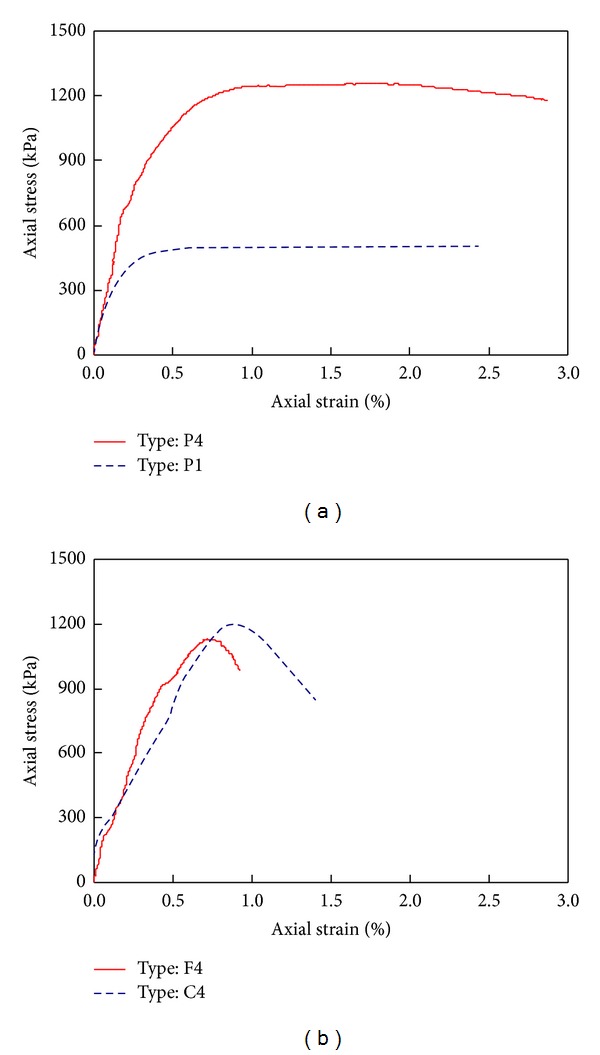
Compressive strength of soil-grout mixtures: (a) specimen without grains; (b) specimen with grains.

Typical stress-strain responses of the grouted soils with and without grains during the axial compression test are plotted in Figure 5. The grouted soils without grains (P-type) showed a ductile behavior after peak strength even though the peak strength of the specimen was dependent on the paste type as shown in Figure 5(a). The peak strengths of type P4 and type P1 were 1200 kPa and 500 kPa, respectively. The strength of the grouted soils increased with a decrease of the water cement ratio. Furthermore, the strains for peak strength were 0.8% for type P4 and 0.4% for type P1. The quick setting agent activates the generation of early hydration products such as ettringite and thaumasite. A concentration of byproducts of soil-grout mixtures renders a decrease in the strength and stiffness of the grout mixture [9]. Thus, the strength and stiffness of soil-grout mixtures was much lower than that of the common cement mixtures.

The grouting without grains (P-type) as shown in Figure 5(a) presented more ductile behavior than those with grains (F-type or C-type) as shown in Figure 5(b). The strengths of the soil-grout specimens with similar mixing ratios were similar (1200 kPa for type C4 and 1100 kPa for type F4) because the unconfined compressive strength is generally related to specimen cohesion, which is induced by the hydration of cement. In addition, the strains for peak strength were also similar (0.8% for type C4 and 0.7% for type F4).

### 4.2. Elastic Waves

**Figure 6:**
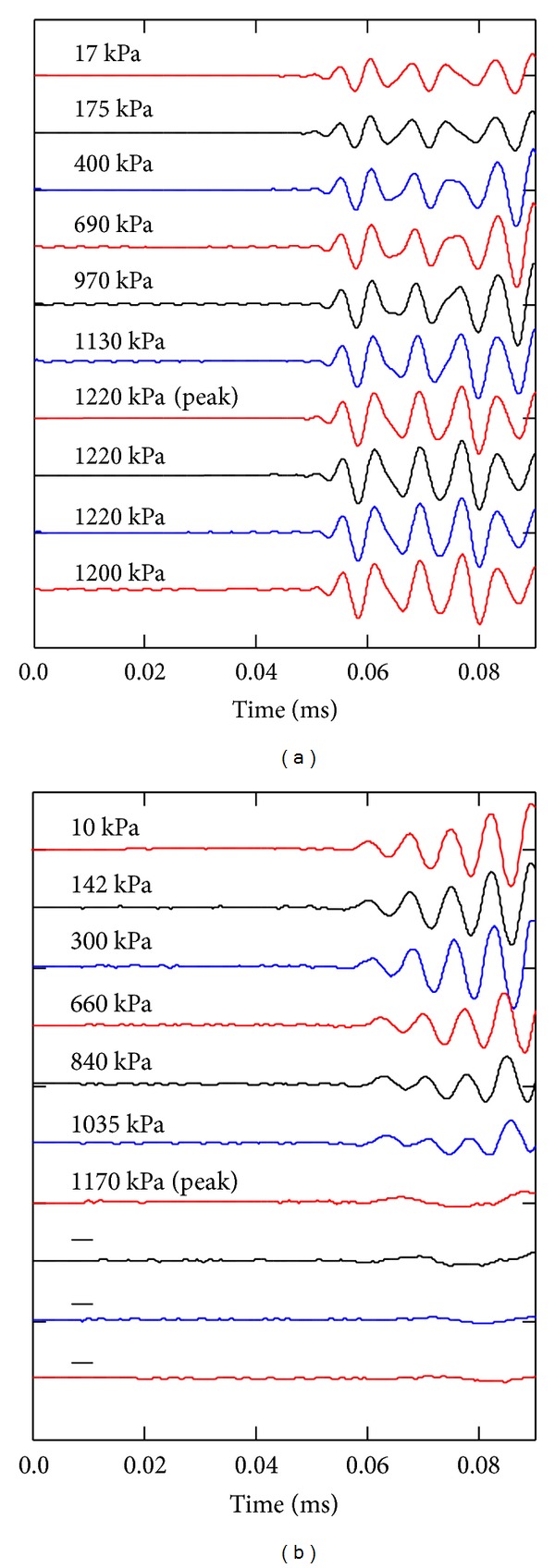
Compressional wave signatures during compressive strength tests for soil-grout mixtures: (a) specimen without grains: Type P4; (b) specimen with coarse grains: Type C4. Numbers on left side at each figure indicate applied axial stress.

Nondestructive tests using compressional waves were conducted during the destructive test (uniaxial compression test). The test procedure for the compressional wave velocity measurements followed the ASTM C597 [10], which described the direct compressional wave velocity measurement. Typical compressional wave responses under applied axial stresses are shown in Figure 6. The first arrival was fairly constant under different axial stresses. The constant first arrival confirmed that the stiffness was less sensitive to the state of stress for the lightly cemented soils [2, 3, 17–19]. As the behavior of the soil-grout mixtures without grains was ductile, the compressional waves were continuously measured after peak strength as shown in Figure 6(a). The grouted soils with grains, however, showed more brittle behavior. Thus, the compressional waves were not measured after peak strength as shown in Figure 6(b). The resonant frequency was 50 kHz for both soil-grout mixtures.

**Figure 7:**
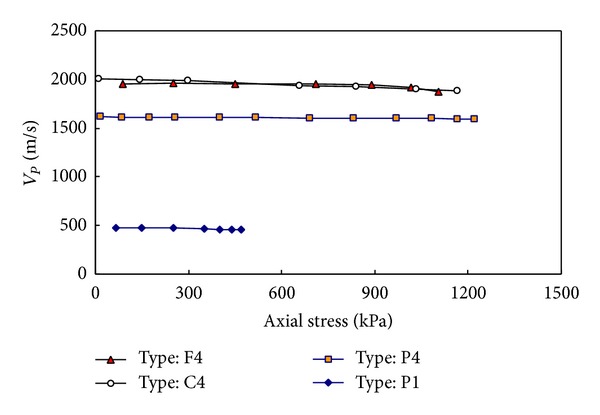
Compressional wave velocity (*V*_*P*_) versus axial stress for different mixture types.

The elastic wave velocities were calculated by using the first arrival and the travel distance of elastic waves. The first arrivals of compressional and shear waves were the first deflection and zero after first bump, respectively [14]. And the travel distances of the elastic waves were the tip-to-tip distance of a pair of transducers [2, 3]. The compressional wave velocities (*V*_*P*_) versus applied axial stress are plotted in Figure 7. Figure 7 shows that the compressional wave velocities of the soil-grout mixtures without grains were almost constant until the mixtures were broken. For the soil-grout mixtures with fine or coarse grains the compressional wave velocities slightly decreased with an increase in the axial stress. Note that as the compressive stress approached the compressive strength, the specimen volume increased (dilatancy behavior) and the elastic wave velocity slightly decreased.

The resonant frequencies of compressional and shear waves are about 50 kHz and 5 Hz, respectively. And the velocities of compressional and shear waves are 550~2240 m/s and 100~230 m/s, respectively. Thus, the wavelengths are 11~45 mm and 20~46 mm and for compressional and shear waves, respectively. Note that the wavelength of the compressional waves for the mixtures with coarse grains ranges from 22 mm to 43 mm. And the median diameters of coarse and fine grains are 3.1 mm and 0.82 mm, respectively (see Table 1). The ratio of the wavelength to the particle size was at least 7, which is greater than the recommended minimum value of 3 in the ASTM D2845 [20], and thus the dispersion, low-pass filtering, and scattering effects may be minor [17].

## 5. Analyses

The compressive strength and stiffness are related to the elastic wave velocities [6]. The nondestructive testing method may be an alternative option for the evaluation of soil-grout improvement.

### 5.1. Compressive Strength versus Compressional Wave Velocity

**Figure 8:**
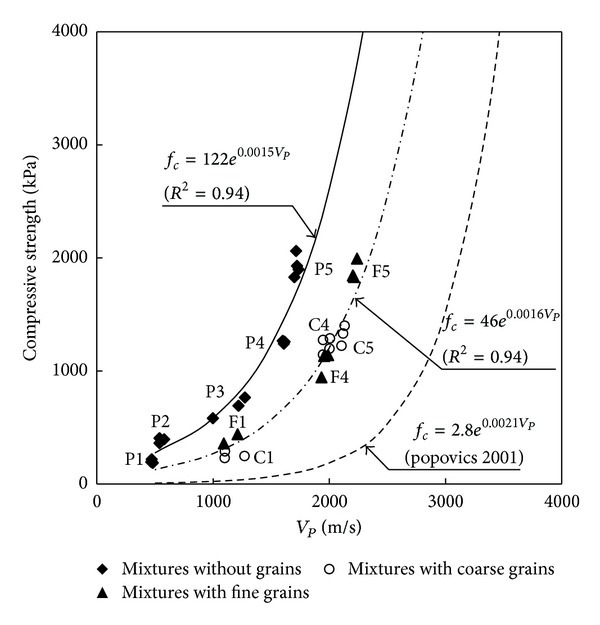
Compressive strength versus compressional wave velocity.

The compressional wave velocities measured in the air were compared with axial compressive strength for all thirty-four specimens. The compressional wave velocity, which was determined at the initial stage of the stress, versus strength is plotted in Figure 8. Figure 8 shows that the axial compressive strength increased with an increase in the compressional wave velocity. The semiempirical exponential functions for the estimation of the axial compressive strength from the compressional wave velocity are superposed in Figure 8. The compressive strength based on the compressional wave velocity for the soil-grout mixtures without grains and the soil-grout mixtures with grains are as follows:

(3) Grouted  mixtures  without  grains  (Type  P):  fc=122e0.0015VPGrouted  mixtures  with  grains  (Type  F  or  C):  fc=46e0.0016VP.

The correlation coefficients of soil-grout mixtures without grains and with grains were greater than 0.94 as shown in Figure 8. The higher values in the correlation coefficients demonstrate that the elastic wave velocity has a strong relationship with the compressive strength.

The mixture ratio of water, cement, and quick setting agent was the same for types P1, F1, and C1 specimens. Specimens with the same mixture ratio (except grains) produced a similar compressive strength, as shown in Figure 8: ~250 kPa for types P1, Fl, and C1 specimens and ~1200 kPa for types P4, F4, and C4 specimens. However, the compressional velocity of type P1 was much smaller than that of types F1 and C1. The difference in the compressional wave velocity for the specimens with the same mixture ratio may result from the different governing factors for the strength and compressional wave velocity. The correlation between strength and compressional velocity in the concrete specimens composed of hardened cement paste, mineral aggregate, and water is also plotted in Figure 8. Figure 8 shows that the *α* values decreased with an increase in the strength, while the *β* values were similar regardless of the strength. The bonding material such as cementation agent controls the compressive strength of soil-grout mixtures. The properties of the grains dominate the compressional wave velocity of soil-grout mixture specimen.

### 5.2. Elastic Modulus versus Compressional Wave Velocity

**Figure 9:**
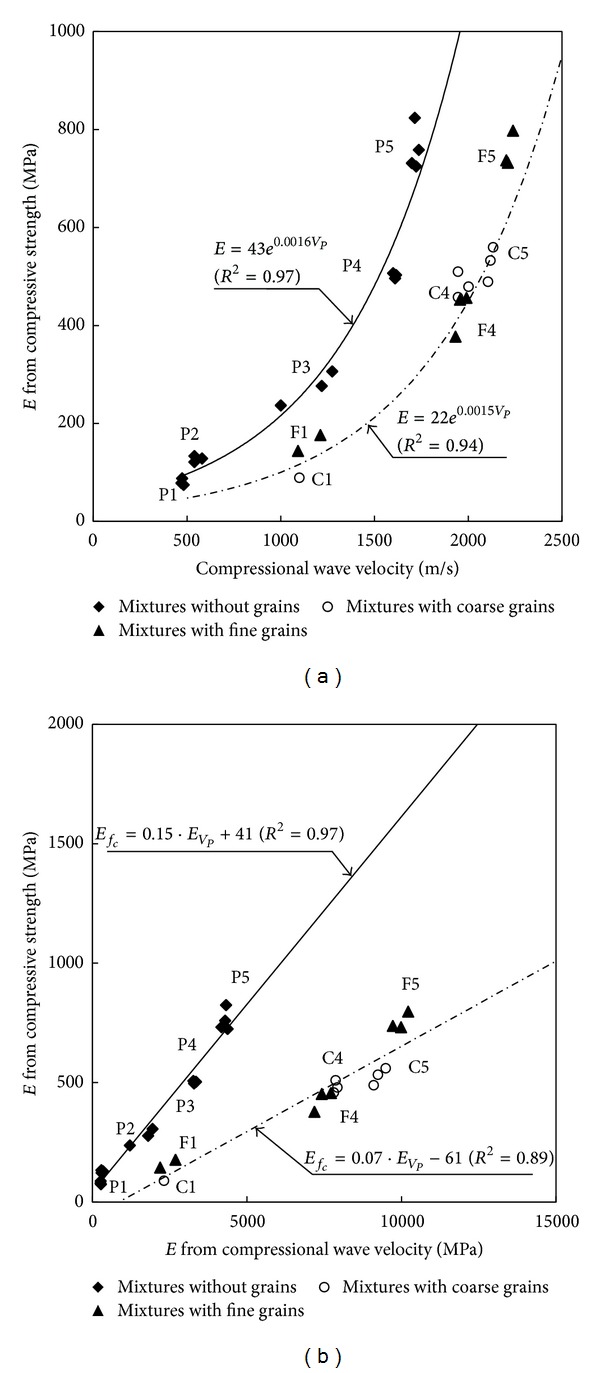
Elastic modulus: (a) elastic modulus measured by axial compression test versus compressional wave velocity; (b) elastic moduli measured by axial compression test versus by compressional wave velocity.

The elastic modulus at large strain was obtained from the destructive compressive strength test. The elastic modulus, *E* [MPa], was also estimated from the compressional wave velocity by using a semiempirical exponential relationship. The results are superposed in Figure 9(a) and the functions are as follows:

(4) Grouted  mixtures  without  grains  (Type  P):  E=43e0.0016VP,Grouted  mixtures  with  grains  (Type  F  or  C):  E=22e0.0015VP.

The trend of the elastic moduli was similar to that of the strength. In addition, the correlation coefficients of soil-grout mixtures without grains and with grains were greater than 0.94. As the slope of the stress-strain curve decreased with an increase in the strain during the axial compression test, the elastic modulus determined at the large strain should be much smaller than the value estimated at the small strain. Note that the strain level, at which the elastic modulus based on the compression wave velocity is determined, is generally less than 10^−4^~10^−3^% [21]. The strain level for the elastic modulus from the axial compression test was about 1%. Thus, the difference in strain levels between compressional wave velocity and static tests is 1,000~10,000 times. This strain gap produces the significant modulus difference between wave propagation method and static failure method as shown in Figure 9(b). In addition, as the ratio of the small strain modulus to the large strain modulus increases with an increase in the stiffness of the specimen [22], the modulus for the mixtures with grains was greater than that for the mixtures without grains as shown in Figure 9(b).

### 5.3. Compressive Strength versus Shear Wave Velocity

**Figure 10:**
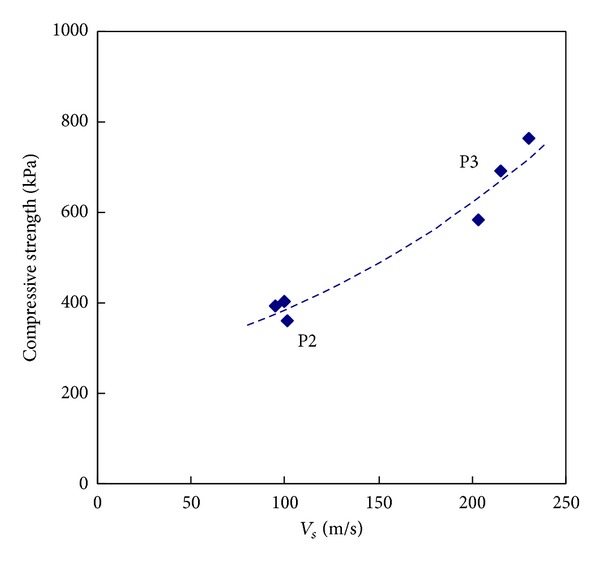
Shear wave velocity versus compressive strength for mixtures without grains.

Shear waves were also monitored during the axial compression test for six soil-grout mixtures without grains (types P2 and P3). The shear waves were measured on the sides across the diameter (rather than at the top and bottom) of the specimens. For the generation and detection of the shear waves, the coupling between the transducers and medium is critical. As the coupling was not perfect, a few cases of the shear waves were only detected. The shear wave velocity was also fairly constant with axial stresses. The shear wave velocities measured in the air versus axial compressive strength is plotted in Figure 10. Figure 10 shows that the shear wave velocities ranged from 100 m/s to 250 m/s, which are reasonable values in lightly cemented soils [2, 3, 17, 23]. Figure 10 shows that the relationship between the compressive strength and the shear wave velocity also followed the exponential function discussed in the compressional wave section.

### 5.4. Compressional Velocity versus Shear Wave Velocity

**Figure 11:**
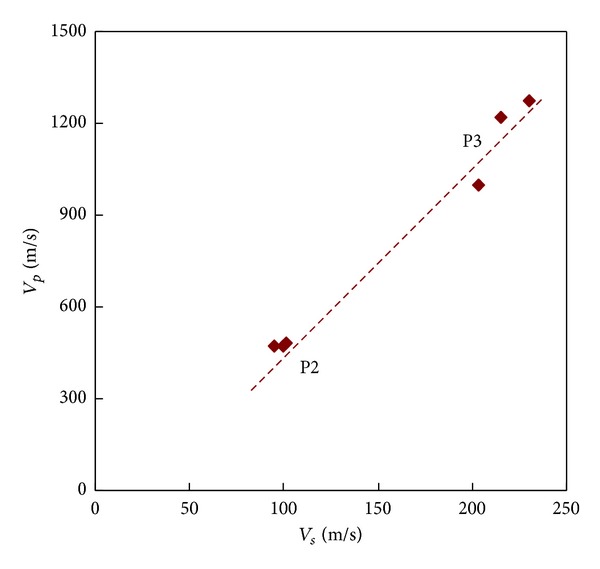
Shear wave velocity versus compressional wave velocity for mixtures without grains.

The compressional wave velocity was compared with the shear wave velocity for six soil-grout mixtures without grains. The linear relationship was observed as shown in Figure 11. The compressional wave velocity was five to six times greater than the shear wave velocity. Based on elasticity, Poisson's ratio *υ* can be calculated as follows:

(5) $$\begin{array}{c} \text{Poisson's}\;\text{ratio:}\;\upsilon =\frac{1}{2}\left(\frac{V_{P}^{2}}{V_{s}^{2}}\right)-1. \end{array}$$

Based on (5), the calculated Poisson's ratio *υ* was about 0.48. Note that during the compressive strength test, water was released from the specimen. The leakage during compressive strength tests confirmed the higher Poisson's ratio.

## 6. Summary and Conclusions

The relationship between the axial compressive strength and elastic wave velocity was investigated by using soil-grout mixtures. Three types of soil-grout mixtures were prepared: grouted mixtures without grains, grouted mixtures with fine grain sands (*D*_sand_ ≈ 0.82 mm), and grouted mixtures with coarse grain sands (*D*_sand_ ≈ 3.1 mm). The uniaxial compressive strength showed a good exponential correlation with the compressional wave velocity. The axial compressive strength versus compressional wave velocity showed that the cementation agent governed the strength at large strain and the grains within soil-grout mixtures controlled the compressional wave velocity at small strain. Exponential models for the estimation of strength and elastic modulus were suggested for the mixtures with and without grains by using the compressional wave velocity. The compressive strength was also correlated to the shear wave velocity. This study demonstrates that the strength and stiffness of the grouted mixtures may be simply estimated by the nondestructive elastic wave velocities.

## Figures and Tables

**Table 1:** Properties of fine and coarse sands.

	Median diameter *D*_50_ (mm)	Coefficient of uniformity *C*_*u*_	Coefficient of curvature *C*_*c*_	Specific gravity *G*_*s*_	Friction angle *ϕ* (deg)
Fine sands	0.82	1.46	0.93	2.62	39~48
Coarse sands	3.10	1.59	0.91	2.63	45~55

**Table 2:** Properties of soil-grout mixtures.

Soil-grout mixtures	Type	Water : cement : agent : sand (weight ratio)	Water-cement ratio	Unit weight (kN/m^3^)	Number of specimens
Mixtures without grains	P1	0.78 : 0.17 : 0.05 : 0.00	4.6	10.3	3
	P2	0.74 : 0.19 : 0.08 : 0.00	3.9	12.0	3
	P3	0.72 : 0.20 : 0.08 : 0.00	3.5	12.1	3
	P4	0.69 : 0.22 : 0.09 : 0.00	3.2	12.8	3
	P5	0.69 : 0.31 : 0.00 : 0.00	2.3	14.5	4

Mixtures with fine grains	F1	0.18 : 0.04 : 0.01 : 0.77	4.6	18.4	2
	F4	0.19 : 0.06 : 0.02 : 0.73	3.2	19.4	3
	F5	0.19 : 0.08 : 0.00 : 0.73	2.3	20.3	3

Mixtures with coarse grains	C1	0.18 : 0.04 : 0.01 : 0.77	4.6	19.3	3
	C4	0.19 : 0.06 : 0.02 : 0.73	3.2	20.7	3
	C5	0.19 : 0.08 : 0.00 : 0.73	2.3	20.8	4

Note: if the typed numbers are the same (e.g., P1, F1, and C1), the mixture ratios of water, cement, and quick setting agent are identical.
